# Open Mouth Posture Syndrome (OMPS): Classification

**DOI:** 10.3390/jcm14103586

**Published:** 2025-05-21

**Authors:** Can-Florian Keleş, David Morais, Anand Marya, Omar Fawzi Chawshli, Adith Venugopal, Ute Ulrike Botzenhart

**Affiliations:** 1 Medical Science (PhD), Faculty of Human Medicine, Paracelsus Medical University, Strubergasse 21, 5020 Salzburg, Austria; ompsyndrome@protonmail.com (C.-F.K.);; 2 University of Puthisastra, Phnom Penh 12211, Cambodia; amarya@puthisastra.edu.kh; 3 Hawler Medical University, Erbil 44001, Iraq; 4 Discipline of Orthodontics, Department of Oral Sciences, Faculty of Dentistry, University of Otago, Dunedin North, Dunedin 9016, New Zealand; 5 Department of Orthodontics and Dentofacial Orthopedics, Dr. D. Y. Patil Dental College and Hospital, Dr. D. Y. Patil Vidyapeeth, Sant-Tukaram Nagar, Pimpri, Pune 411018, India; 6 Medical Faculty Carl Gustav Carus, TU Dresden, 01307 Dresden, Germany; 7 University of Medical Sciences, 60-812 Poznań, Poland

**Keywords:** mouth breathing, craniofacial development, tongue-related pathologies, malocclusion, open-mouth posture syndrome, airway obstruction, oral posture dysfunction, myofunctional disorders, orofacial imbalance, interdisciplinary orthodontics

## Abstract

**Objectives**: This narrative review aims to redefine Open Mouth Posture Syndrome (OMPS) as a multifactorial condition with overlapping symptoms and a cyclical pathophysiology. A novel classification system for OMPS subtypes is proposed to standardize research approaches and enhance clinical understanding. **Methods**: An interdisciplinary literature review was conducted, focusing on structural, functional, and adaptive mechanisms underlying OMPS. Subtype definitions were refined based on recent findings. **Results**: OMPS is categorized into five subtypes: Obstructive, Habitual, Anatomical, Sleep-Disordered Breathing, and Tongue-Related Pathologies. These subtypes share interconnected etiologies and manifestations, contributing to a feedback loop that complicates diagnosis and management. **Conclusions**: This classification system lays the foundation for future research and clinical protocols, emphasizing the need for a systematic approach to understanding OMPS.

## 1. Introduction

*Open Mouth Posture Syndrome* (OMPS) represents a complex physiological condition characterized by deviations in oral posture that fundamentally disrupt orofacial equilibrium. The syndrome emerges from an intricate interplay of structural, functional, and adaptive mechanisms that progressively impact multiple physiological systems [1].

OMPS can be defined and is documented as an oral postural defect that is based on the tongue adopting a pathological posture.

**Figure 1:**
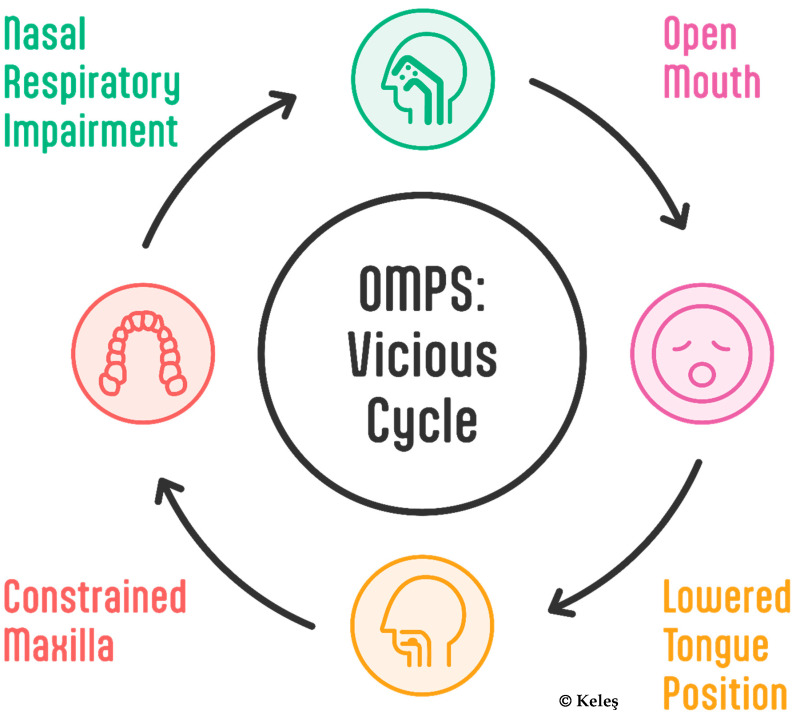
The vicious cycle of OMPS: Nasal respiratory impairment (green) leads to oral breathing and, thus, an open mouth (pink). This in turn leads to a lowered tongue position (orange), and the absence of a stimulus on the maxilla constrains the jaw (red), which completes the cycle by impairing nasal respiration.

This syndrome’s defining characteristic is its cyclical nature (see Figure 1), where initial functional disruptions trigger cascading structural adaptations. This feedback loop creates a complex pathological process that obscures traditional cause-and-effect relationships.

The key physiological dynamics of this condition involve disrupted nasal respiration, which, along with tongue-related postural pathologies, triggers compensatory mechanisms. These postural defects may also stem from functional immobility of the lingual fascia. As a result, the muscular and neurological systems become progressively intertwined, leading to altered craniofacial development and, consequently, malocclusion. These structural changes further compromise respiratory function.

Historically, the broader term *Mouth-Breathing Syndrome* (MBS) inadequately captured the syndrome’s complexity, leading to low certainty in the evidence of its implications. OMPS introduces a more nuanced framework, distinguishing five subtypes to capture the multifaceted nature of oral posture disorders.

The cyclical interplay of structural abnormalities, functional deficits, and compensatory mechanisms creates a feedback loop that challenges traditional diagnostic approaches.

For instance, chronic mouth breathing due to nasal obstruction may alter craniofacial development, generate symptoms such as malocclusions, exacerbate respiratory dysfunction, and simultaneously have a systematic impact on the body’s posture, potentially in association with inflammatory markers [2].

This intricate progression blurs distinctions between primary and secondary pathologies, necessitating a comprehensive, interdisciplinary approach to understanding and managing OMPS [3,4,5,6].

### 1.1. From MBS to OMPS

Nasal breathing (NB) represents the physiological norm, essential for proper craniofacial growth and development. Mouth breathing (MB) fundamentally disrupts this delicate balance, leading to progressive alterations in craniofacial structures and respiratory function [4,5,6].

The etiology of malocclusions remains significantly under-investigated in orthodontics [7], highlighting the need for more precise investigative tools [6,8,9,10]. Current research often overlooks critical signs of OMPS, particularly tongue-related pathologies, impeding systematic understanding. This has not been improved since the introduction of the modern MBS classification by *Sam and Finn* (1987) [11].

#### 1.1.1. MBS Classification Framework

MB is defined as breathing through the mouth 5–30% of the time for longer than six months [6,12,13,14,15].

This condition has different causes:

- Obstructive MB

This form is caused by physical obstructions within the airway that encourage compensatory oral breathing.

- Habitual MB

This form consists of a persistent open oral posture despite the removal of obstructions, often resulting from chronic habits or open lip posture.

- Anatomical MB

This is the inability to seal one’s lips due to structural limitations, such as lip incompetence.

#### 1.1.2. Topography of Obstruction

**Figure 2:**
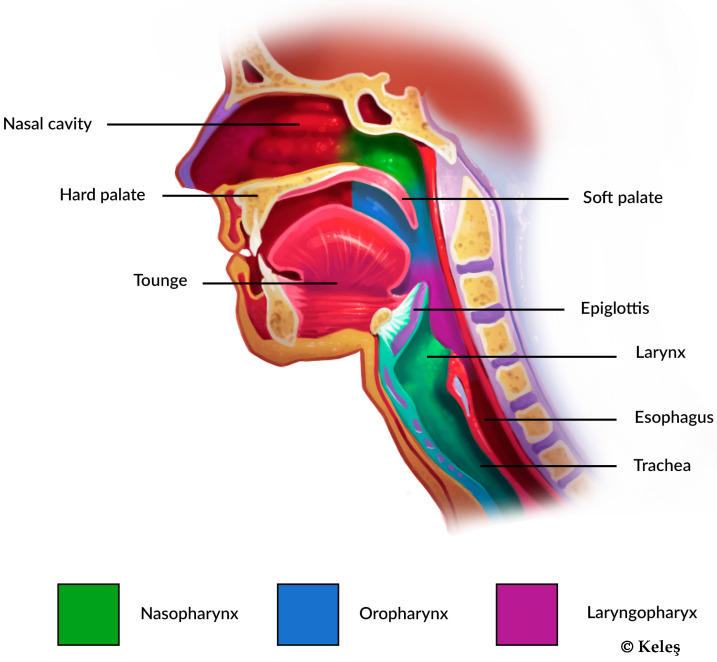
Cross section of a human head. The upper airway, which can be divided into nasopharynx (green), oropharynx (blue), and laryngopharynx (purple).

Investigations for Obstructive MB can be subcategorized even further based on the topography of the upper airway, specifically with respect to the nasal cavity, nasopharynx, oropharynx, or laryngopharynx (see Figure 2), leading to different chances of phenotypical expression [6]. Similarly, asthma and other Chronic Obstructive Pulmonary Diseases (COPDs) affect the lower airway: the trachea, bronchioles, and alveoli [16].

#### 1.1.3. Sleep-Disordered Breathing

Sleep-Disordered Breathing (SDB) seems to be an additional, special sub-classification, comprising the subcategories of snoring [9,15,17,18], Obstructive Sleep Apnea, and Central Sleep Apnea [19]. Adequate oxygenation via nasal respiration is critical for cognitive function and restorative sleep [20]. But chronic MB interferes with these processes, disrupting a regulated, rhythmic intake of airflow [21]. This results in the loss of valuable information required for a proper, emotionally regulated, functioning lung circuit [22] “due to a combination of unsteady sleep and instability of the central respiratory drive” [23], possibly resulting in reduced growth hormone (GH) [24] release during deep sleep. This disruption stunts growth and cognitive development in children [25]. Thus, “[many] children with sleep disorders are often well below their peers in terms of height and weight” [26], all the while impacting learning ability and cognitive skills [3,27,28,29,30,31,32,33].

#### 1.1.4. Tongue-Related Issues

MBS, by definition, requires MB and thus an open lip posture [8,34]. But similar changes to facial growth are associated with tongue-related issues, such as ankyloglossia or a lower resting position of the tongue [35,36,37,38,39]. Therefore, the basis of most OMPS symptoms is oral imbalance due to its pathological altered tongue position/dyskinesia even when the lips are sealed [8,9,36,40,41,42,43,44].

Remarkably, 9 out of 10 adolescents with dentofacial anomalies exhibit orofacial dysfunctions, including an open mouth posture (OMP), a pathological tongue rest position, visceral swallowing, and articulation disorder [45]. But formation adapts to usage. Thus, it is structurally understandable that ankyloglossia, among others, potentiates the collapse of the oropharyngeal airway during sleep, increasing the risk of developing SDB [35]. But the extreme impact of tongue positioning and its function cannot be overstated. Therefore, recent classification systems have further refined our understanding of tongue functional impairments, subdividing etiological classifications into anterior, posterior [44], and mid-tongue mobility restrictions.

## 2. Materials and Methods

This narrative review was conducted to explore the current knowledge on and multifactorial nature of Open Mouth Posture Syndrome (OMPS) and propose a novel classification framework based on emerging interdisciplinary insights. This review was conducted according to a structured yet flexible approach consistent with narrative review methodology.

### 2.1. Literature Search Strategy

A comprehensive literature search was performed using the PubMed, Google Scholar, and Scopus databases. In the initial search, we employed the MeSH term “mouth breathing”, combined with relevant keywords such as “facial development”, “craniofacial growth”, “oral posture”, and “malocclusion”. To expand the scope and capture syndromic overlaps, subsequent searches included MeSH terms like “oronasal breathing”, “impaired nasal respiration”, “mouth breathing diagnosis”, “tongue resting position” and “ankyloglossa”.

The primary focus was on research published up to the end of 2023. Articles published after this cut-off were not included in depth, as the classification system was developed based on data and insights available at that time.

### 2.2. Inclusion and Exclusion Criteria

The inclusion criteria encompassed peer-reviewed articles, original research papers, systematic and narrative reviews, and meta-analyses that addressed topics such as mouth breathing, orofacial development, nasal obstruction, and tongue posture. Studies were considered if they explored structural, functional, neurological, and postural aspects within the fields of orthodontics, otolaryngology, sleep medicine, and myofunctional sciences. Both English-language publications and selected non-English-language work with relevant methodological or conceptual value were included.

In contrast, the exclusion criteria ruled out case reports, anecdotal observations, non-peer-reviewed publications, grey literature, and studies lacking a clear conceptual or diagnostic reference to oral or respiratory posture.

### 2.3. Literature Selection and Processing

Approximately 500 relevant publications were included and thematically analyzed for this review. The selection emphasized studies with clear methodological frameworks, classification models, or definitional clarity. Particular attention was paid to how tongue posture, airway obstruction, and compensatory mechanisms were operationalized within each study. While the majority of the literature reviewed was in English (~87%), additional publications included five in German, three in Portuguese, two in French, two in Chinese, and one in Hungarian. Non-English articles were translated using DeepL translation services (https://www.deepl.com/de/translator/, accessed on 30 April 2025) and reviewed by native speakers to ensure accuracy and contextual consistency.

### 2.4. Conceptual Synthesis

Due to the conceptual nature of this narrative review and the heterogeneity of the sources, no systematic data extraction or meta-analysis was conducted. Instead, key thematic patterns, recurring syndromic definitions, and physiological frameworks were identified and consolidated.

During the synthesis phase, it became evident that existing models—particularly those based on static or binary definitions of active breathing modes—failed to capture the dynamic, multifactorial character of OMPS. The lack of objectivity and reproducibility in describing tongue rest position or subtle oral postural deviations highlighted the need for a refined framework. This recognition led to a collaborative interdisciplinary consultation process, drawing on clinical experience and emerging theoretical models. These efforts culminated in the development of a novel classification scheme, grounded in recent findings and cross-disciplinary dialogue, to better conceptualize and categorize OMPS in clinical and research contexts.

## 3. Results

### 3.1. Common Patterns Across Categories

**Formation Adapts to Usage**

Chronic, subtle biomechanical forces progressively reshape physiological structures. Oral functions—respiration, suckling, swallowing, mastication, and speech—depend on a delicate physiological balance. Disruptions in mandibular, tongue, or head posture initiate cascading abnormal growth patterns in soft and hard tissues [5,34,46,47,48] since “the maxillary response is mainly determined by tongue posture and movements” [6,49].

**Fundamental biomechanical principles:**

- Weak, persistent forces induce gradual tissue adaptation.
- Individual variations significantly impact physiological responses [5].
- Compensatory mechanisms emerge to maintain functional equilibrium [6].

**Typical changes associated with this syndrome:**

***Skeletal Changes: vertical growth predominance*** [34,50,51,52]

Development of “long face syndrome” or “adenoid face” (coined by *Tomes* in 1872) [46];

- Convex facial type [8,53].
- Increased lower facial height, steeper mandibular plane [54,55,56], downward rotation of the mandible [50,52,53];
- Protruded upper lip [51,53];
- Longer lower lip [51];
- Obtuse nasal angle [8,51,53,57,58];
- Fattened philtrum [50];
- Reduced facial attractiveness [26,59];
- Decrease in nose prominence [50,53], with “disuse atrophy” of the lower lateral cartilages [46,50];
- Open lips/open mouth [8,34,60,61,62,63].

***Dental Effects: Malocclusion Patterns*** [5,8]***:***

- Anterior open bite tendency [8,10,49,64,65,66,67];
- Increased overjet [8,10,49,55,56,64,65,66,67];
- Posterior crossbite risk [8,10,49,64,65,66,67] due to constricted maxilla [54,68], often resulting in a narrow maxillary arch with a high palate [8,10,49,52,69];
- Sagittal discrepancies [5,8].

***Functional Impacts*** ***:***

- Reduced nasal breathing efficiency increases breathing effort, thus altering breathing patterns and restricting airflow [46];
- Speech pathology [9,45,64].

*Muscular Adaptations:*

- Modified tongue position [9,68];
- Altered perioral muscle activity [68];
- Distinct eating habits [70];
- Postural compensations—a forward Head Posture (FHP) emerges as a compensatory airway expansion mechanism [71,72,73,74,75];
- Compensations include not only the cervical part of the spine but the whole body [71].

***Systemic Effects***

*Sleep Quality*

- Disrupted sleep patterns [15];
- Possible systemic physiological changes [26,76,77];
- Daytime consequences (*see Quality of Life*).

*Quality of Life* [12,15,78,79,80]

- Behavioral changes, social implications, and academic/cognitive impacts [3,26,27,28,29,30,31,32,81,82].

***Key Observations***

- All the subcategories show remarkable overlap in their effects on craniofacial development.
- The timing of onset appears to influence severity.
- Early intervention is crucial across all categories.
- Multiple systems are affected regardless of the initial cause.
- Treatment requires a comprehensive approach due to shared impacts.

**Figure 3:**
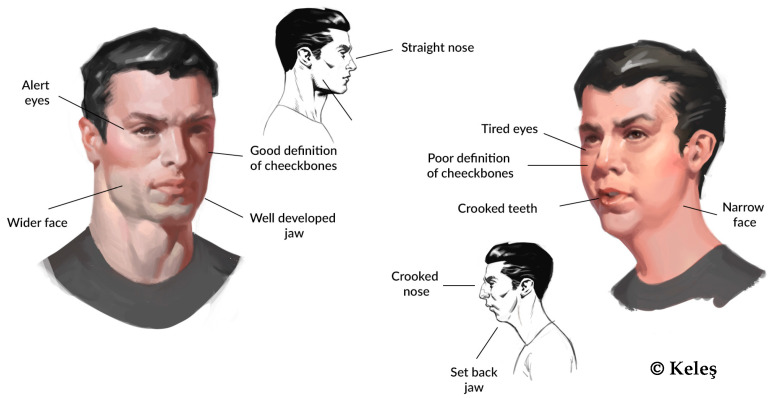
OMPS extraoral signs—a potential expression of a healthy physiological nose breather versus a phenotypical expression of a mouth breather.

Mouth breathers characteristically exhibit distinctive craniofacial morphological changes (see Figure 3).

While the periosteal matrices (tongue and teeth) directly influence the skeletal unit—according to Moss’s matrix theory—proper NB function (capsular matrices: functioning oronasopharyngeal space) is indirectly necessary for a face to grow healthy [83]. “It appeared that, under the pressure of the respiratory drive, each animal would find its own most convenient way to secure the oral airflow and then develop a dental malocclusion in accordance with this new function” [5], highlighting physiological complexity. The intricate interplay between respiratory function, muscular adaptation, and skeletal growth defies simplistic linear explanations. Each individual’s compensatory mechanisms represent a unique biomechanical signature [6].

### 3.2. OMPS Classification Framework

The understanding of MBS evolved with the investigative nature of the scientific method over time. But its closely shared impact on craniofacial development obscures its clear impact when compared to non-mouth breathers. Due to recent advances in methodology and the researched impact on craniofacial development and physiology, the need to breathe through the mouth is no longer required to explain its impact. Therefore, a more concise and extensive term encompasses a central figure, the tongue, which shapes the oral cavity and thus craniofacial development, hence Open Mouth Posture Syndrome (OMPS).

**Figure 4:**
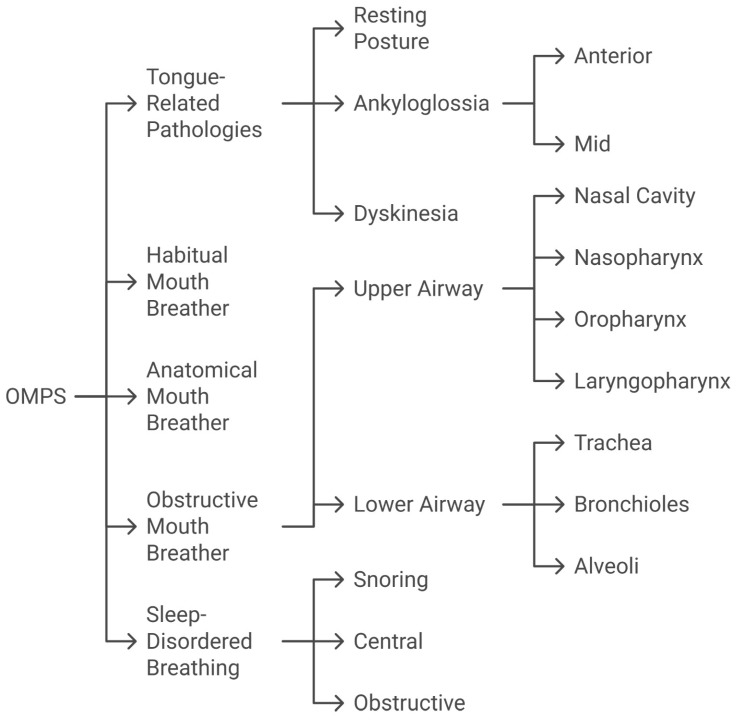
Flow chart of OMPS classification with its 5 subtypes and further subdivision.

The proposed classification framework categorizes OMPS into five distinct subtypes: Obstructive, Habitual, Anatomical, Sleep-Disordered Breathing, and Tongue-Related Pathologies (see Table 1 and Figure 4). These subtypes reflect overlapping etiologies and manifestations, forming a complex feedback loop that complicates both diagnosis and treatment. Each subtype is characterized by specific structural, functional, and adaptive mechanisms that contribute to the syndrome’s progression. By systematically delineating these categories, this classification provides a foundation for targeted research on and improved clinical management of OMPS.

## 4. Discussion

**The Need for Classification**

The current healthcare landscape reveals a critical gap in understanding and addressing OMPS [26]. Dentists, often the first professionals to encounter patients with these symptoms, must be equipped to recognize and intervene early [26,34,84].

***Strategic Significance of OMPS Classification:***

*1*. *Educational advancement*
  - It provides educators with a comprehensive framework for teaching others about the etiological aspects of malocclusion.
  - It enhances our understanding of developmental processes in oral health.
  - It bridges gaps between interdisciplinary medical approaches.

*2*. *Research Imperatives*

- This classification enables the systematic investigation of varying risks and symptom expressions.
- It facilitates the analysis of correlations between severity, symptom variability, and etiological factors.
- It addresses the stagnant classification system unchanged since *Sam and Finn’s* work 40 years ago [11,85].

*3*. *Comprehensive Research Objectives Future research should explore* [6]:

- The genetic factors influencing OMPS;
- Environmental determinants of malocclusion;
- The development of validated diagnostic criteria;
- Interdisciplinary treatment protocols.

***Methodological Innovations—The OMPS classification system represents a paradigm shift in understanding oral posture disorders. By delineating subtypes, this framework allows for the following:***

- The identification of subtype-specific impacts on craniofacial growth;
- The standardization of data collection for precise analyses (*see subsequent article for diagnostics*).
- The facilitation of the development of tailored diagnostic and therapeutic protocols (*see subsequent article for diagnostics*).

***Limitations and future directions—While the classification offers significant insights, it also acknowledges the complexity of OMPS:***

- Management requires individualized, interdisciplinary approaches.
- Subsequent research will focus on developing practical diagnostic protocols.
- Ongoing refinement of the classification is anticipated.

***Broader implications—The OMPS classification transcends traditional diagnostic boundaries, offering the following:***

- A holistic view of oral posture disorders;
- Interdisciplinary research opportunities;
- Potential for personalized intervention strategies.

## 5. Conclusions

The term MBS is not sufficient for describing either etiological changes in craniofacial structures or postural misalignments. But the phenotypical expressions can now be better understood due to recent scientific developments, including subcategories. Therefore, the term OMPS has led to a more intuitive and holistic understanding of malformations, impacting most citizens in the industrialized world and almost every orthodontic patient. OMPS encapsulates a spectrum of interconnected disorders that necessitate subclassification to fully reveal their cyclical nature. The proposed classification provides a foundational framework for comprehensive systematic investigation.

***Key Contributions:***

- A systematic approach to understanding the syndrome complex was developed.
- A quantitative framework for analyzing odds ratios was provided.
- A detailed exploration of severity variations across subtypes was conducted.

The accompanying article in this series will delve into diagnostic protocols, providing practical frameworks for scientific investigations and clinical applications.

The OMPS classification represents not only a taxonomic exercise but a critical step towards understanding the complex interplay of factors governing oral posture, systemic implications, and craniofacial development.

## Figures and Tables

**Table 1:** Overview of OMPS categories.

I. Tongue-related pathologies		
	A. Resting posture (habit)	
	B. Ankyloglossia (mobility impairment)	
	C. Dyskinesia (e.g., reverse swallowing)	
II. Habitual Mouth Breather		
III. Anatomical Mouth Breather		
	A. Lip incompetence	
IV. Obstructive Mouth Breather		
	A. Upper airway:	
		Nasal cavity
		2. Nasopharynx
		3. Oropharynx
		4. Laryngopharynx
	B. Lower airway:	
		5. Trachea
		6. Bronchioles
		7. Alveoli
V. Sleep-disordered breathing		
	Snoring	
	B. Central	
	C. Obstructive	

OMPS classification (types and subtypes).

## Data Availability

The full dataset derived from the literature is available from the first author.

## Resources

Microsoft Word 2021 and EndNote 20 were used to format and create references for this article. Upon conceptualizing Figure 1 with Napkin AI V3, In Design and Photoshop were used to enhance visualization. Figure 2 and Figure 3 were artistically designed in Photoshop 2023. Figure 4 was constructed using Napkin AI. Copyrights for all the figures and intellectual property are held by Can-Florian Keleş.
